# Chronic disease multimorbidity and mental health among older adults in China: The moderating roles of family and social support

**DOI:** 10.1371/journal.pone.0357259

**Published:** 2026-09-10

**Authors:** Ling Zong, Kexin Zhou

**Affiliations:** 1 School of Health, Jiangsu Vocational Institute of Commerce, Nanjing, Jiangsu, China; 2 National Research Center for Resettlement, Hohai University, Nanjing, Jiangsu, China; Nanjing University, CHINA

## Abstract

Population aging has been accompanied by a growing prevalence of chronic disease multimorbidity among older adults, raising increasing concerns about its implications for mental health. Although previous studies have documented the negative association between multimorbidity and mental health, evidence regarding how support systems influence this relationship remains limited. Using nationally representative cross-sectional data from the 2020 China Longitudinal Aging Social Survey (CLASS), this study examined the association between self-reported multimorbidity and mental health among older adults in China, with particular attention to the moderating roles of family support and social support. Multivariate regression models incorporating interaction terms were employed to estimate both direct and interaction effects. The results indicate that, first, multimorbidity is significantly associated with poorer mental health among older adults. Second, although both social support and family support are positively associated with better mental health, only family support significantly moderates the association between multimorbidity and mental health. Third, marginal effects analysis shows that the negative association between multimorbidity and mental health becomes progressively stronger as the level of family support increases. Fourth, subgroup analysis reveals that the direct association between family support and mental health follows a U-shaped pattern across age groups, whereas its moderating effect on the relationship between multimorbidity and mental health gradually weakens with advancing age and becomes statistically insignificant among adults aged 80 years and above. These findings contribute to a more nuanced understanding of interpersonal resources in later life and highlight the need to integrate family-centered approaches into geriatric care and mental health interventions for older adults living with multimorbidity.

## Introduction

Population aging has accelerated worldwide, making chronic non-communicable diseases one of the most important public health challenges affecting the health and well-being of older adults. The World Health Organization defines multimorbidity as the coexistence of two or more chronic conditions within the same individual [1]. Owing to the long-term, cumulative, and often irreversible nature of chronic diseases, multimorbidity not only increases the risks of functional disability, reduced quality of life, and healthcare utilization but also imposes persistent psychological burdens through continuous disease management, functional limitations, and reduced independence [2]. As population aging continues, multimorbidity has become increasingly common among older adults, making its psychological consequences an important issue for both researchers and policymakers.

Multimorbidity is highly prevalent among older populations worldwide. For example, among U.S. adults with a mean age of 48.5 years, 51.0% of women have at least two cardiometabolic or kidney diseases, and 1.5% have three or more such conditions. Among older adults in the United States, the prevalence of multimorbidity reaches approximately 77.0%, while more than half of individuals aged 65–74 years live with multiple chronic conditions. In the United Kingdom, over 70% of adults aged 75 years and older experience multimorbidity [3]. China has likewise entered an era of rapid population aging, accompanied by a substantial increase in the prevalence of chronic diseases among older adults. National survey data indicate that 86.33% of community-dwelling older Chinese adults report having at least one chronic disease [4]. A growing body of evidence from both developed and developing countries consistently demonstrates that increasing numbers of chronic conditions are associated with higher levels of depression and anxiety, poorer subjective well-being, and lower psychological quality of life. Reflecting these concerns, the United Nations 2030 Agenda for Sustainable Development identifies the promotion of healthy lives and well-being for all ages (SDG 3) as a global priority, recognizing mental health in later life as an essential component of healthy aging and sustainable development [5]. Despite extensive evidence documenting the adverse association between multimorbidity and mental health, considerably less attention has been paid to the psychosocial resources that may alter this relationship [6].

To explain why the psychological consequences of multimorbidity differ across individuals, health psychology research has increasingly adopted the Stress-Buffering Hypothesis, which posits that psychosocial resources can intervene in the stress process and reduce the detrimental effects of stressful life events on mental health [7]. Within this framework, chronic disease burden represents a persistent health-related stressor, whereas informal support systems constitute important coping resources that may influence how individuals respond to disease-related challenges [8]. Consequently, the association between multimorbidity and mental health may vary according to the availability of supportive interpersonal resources.

Importantly, informal support is not a homogeneous construct. According to Social Convoy Theory, individuals are surrounded by a dynamic network of social relationships organized into concentric circles according to emotional closeness and relationship quality [9]. The innermost circle primarily consists of family members, whereas the outer circles include friends, neighbors, and community members. Because these sources of support differ substantially in emotional intimacy, caregiving responsibility, and interaction frequency, they may exert distinct influences on psychological adaptation to chronic disease. Accordingly, this study conceptualizes informal support as two separate dimensions. Family support refers to emotional, instrumental, and financial assistance provided by close family members, particularly spouses and adult children, and is characterized by long-term commitment and intergenerational obligations. Social support, by contrast, refers to assistance obtained from broader interpersonal networks, including friends, neighbors, and community members. These operational definitions distinguish interpersonal support from broader sociological constructs such as generalized social capital or social participation [10].

This distinction is particularly relevant in the Chinese context. Although China is experiencing rapid population aging and continuous expansion of community-based support services, the family remains the cornerstone of elder care because of longstanding cultural norms emphasizing filial responsibility. Family support and broader social support therefore coexist but may play different roles in helping older adults cope with chronic disease. Nevertheless, existing evidence remains limited regarding whether these two forms of informal support differentially modify the association between multimorbidity and mental health. Moreover, little is known about whether these associations vary across different levels of support or across different stages of later life.

To address these gaps, this study uses nationally representative data from the 2020 China Longitudinal Aging Social Survey (CLASS) to examine the association between self-reported multimorbidity and mental health among older adults in China. Specifically, we distinguish between family support and social support to investigate their respective moderating roles in the relationship between multimorbidity and mental health. Furthermore, marginal effects analyses are conducted to examine how the association between multimorbidity and mental health changes across different levels of support, while age-stratified analyses explore whether the moderating role of family support differs among adults aged 60–69 years, 70–79 years, and 80 years and above.

This study contributes to the literature in three important ways. First, by distinguishing family support from social support, it extends the application of the Stress-Buffering Hypothesis and Social Convoy Theory to the study of multimorbidity and mental health among older adults. Second, beyond testing interaction terms, it employs marginal effects analyses to provide a more nuanced interpretation of how family support shapes the association between multimorbidity and mental health. Third, the age-stratified regression analysis indicates that the moderating association of family support differs across age groups, suggesting that the contribution of informal support may vary among older adults at different ages. These findings provide evidence for developing more targeted family-centered strategies to support healthy aging and psychological well-being among older adults living with multimorbidity.

The remainder of this paper is organized as follows. The Literature Review and Analytical Framework section reviews the relevant literature and develops the research hypotheses. The Materials and Methods section describes the data, variables, and methodology. The Results section presents the empirical findings. The Discussion section discusses the main findings and their implications. The Conclusions section summarizes the main findings and policy implications. Finally, the Limitations and Future Work section outlines the study limitations and directions for future research.

## Literature review and analytical framework

### Literature review

#### Multimorbidity and mental health in older adults.

With the rapid acceleration of global population ageing, chronic disease multimorbidity has become the norm rather than the exception among older adults. The World Health Organization identifies multimorbidity as a central challenge to healthy ageing (WHO, 2022) [8]. Empirical evidence shows that hypertension, diabetes, arthritis, and cardio-cerebrovascular diseases are the most prevalent and commonly co-occurring chronic conditions in later life [1]. The burden of multimorbidity extends well beyond the simple accumulation of diseases, generating synergistic and compounding health effects [11].

A substantial body of research demonstrates that multimorbidity is a major risk factor for mental health problems such as depression and anxiety [12,13], and is strongly associated with more severe depressive symptoms, lower subjective well-being, and poorer overall mental health [14]. Certain chronic conditions (e.g., cerebrovascular diseases and thyroid disorders) are closely linked to neuroendocrine changes that may contribute to emotional disorders [15]. Moreover, declines in functional capacity, loss of social roles, and heightened uncertainty about future health can significantly undermine self-efficacy and increase feelings of stigma, thereby exacerbating psychological distress [16]. Consequently, the relationship between multimorbidity and mental health reflects a complex process shaped by biological, psychological, and social factors.

#### Social support and mental health.

Social support has long been recognized as a critical psychosocial resource for mental health [17]. Classical social support theory suggests that emotional, instrumental, and informational support are associated with greater perceived security, lower stress levels, and more adaptive coping behaviors, thereby contributing to better psychological well-being [9]. Empirical studies consistently show that higher levels of social support are related to lower risks of depression, greater life satisfaction, and better subjective well-being, both in the general population and among older adults [18]. In addition, social participation, neighborhood interactions, and access to community services have been found to show significant positive correlations with mental health in later life [19].

However, most existing studies treat “social support” as a homogeneous construct. According to the Social Convoy Theory (Antonucci, 1986), informal support systems are structured in concentric circles [9]. This perspective highlights the necessity to distinguish between family support (the inner circle)—typically rooted in kinship, emotional bonds, and normative obligations, and characterized by greater stability and intimacy—and broader social support (the outer circles), which is often more instrumental, selective, and geographically or interest-based [10]. In China, where Confucian values emphasize familial responsibility and intergenerational reciprocity, the family remains the primary unit of welfare provision, and family support plays an irreplaceable role in health protection for older adults [20]. Despite this, empirical research that systematically compares the relative moderating roles of family support and social support on the mental health consequences of multimorbidity among Chinese older adults remains scarce.

#### Literature summary and research gaps.

While the literature has reached broad consensus on the negative associations between chronic disease multimorbidity and older adults’ mental health, significant gaps remain regarding how different sources of support interact with this relationship. Specifically, few studies have distinguished between family support and social support within a unified analytical framework or examined their heterogeneous moderating patterns based on cross-sectional data from the Chinese context.

### Research hypotheses

To address these gaps, this study draws upon the Stress-Buffering Hypothesis, which suggests that psychosocial resources may influence the relationship between health-related stressors and psychological well-being. Multimorbidity represents a major health stressor in later life, whereas informal support systems may shape how older adults experience and respond to this burden. Based on this theoretical framework, the following hypotheses are proposed:

**Hypothesis 1 (H1):** Self-reported multimorbidity is negatively associated with mental health among older adults.

Furthermore, according to the Convoy Model of Social Relations, family members and broader social networks occupy different positions within older adults’ support systems and may therefore play distinct roles in the relationship between multimorbidity and mental health. Accordingly, we propose the following hypotheses:

**Hypothesis 2 (H2):** Social support moderates the association between multimorbidity and mental health.
**Hypothesis 3 (H3):** Family support moderates the association between multimorbidity and mental health.

### Analytical framework

**Fig 1:**
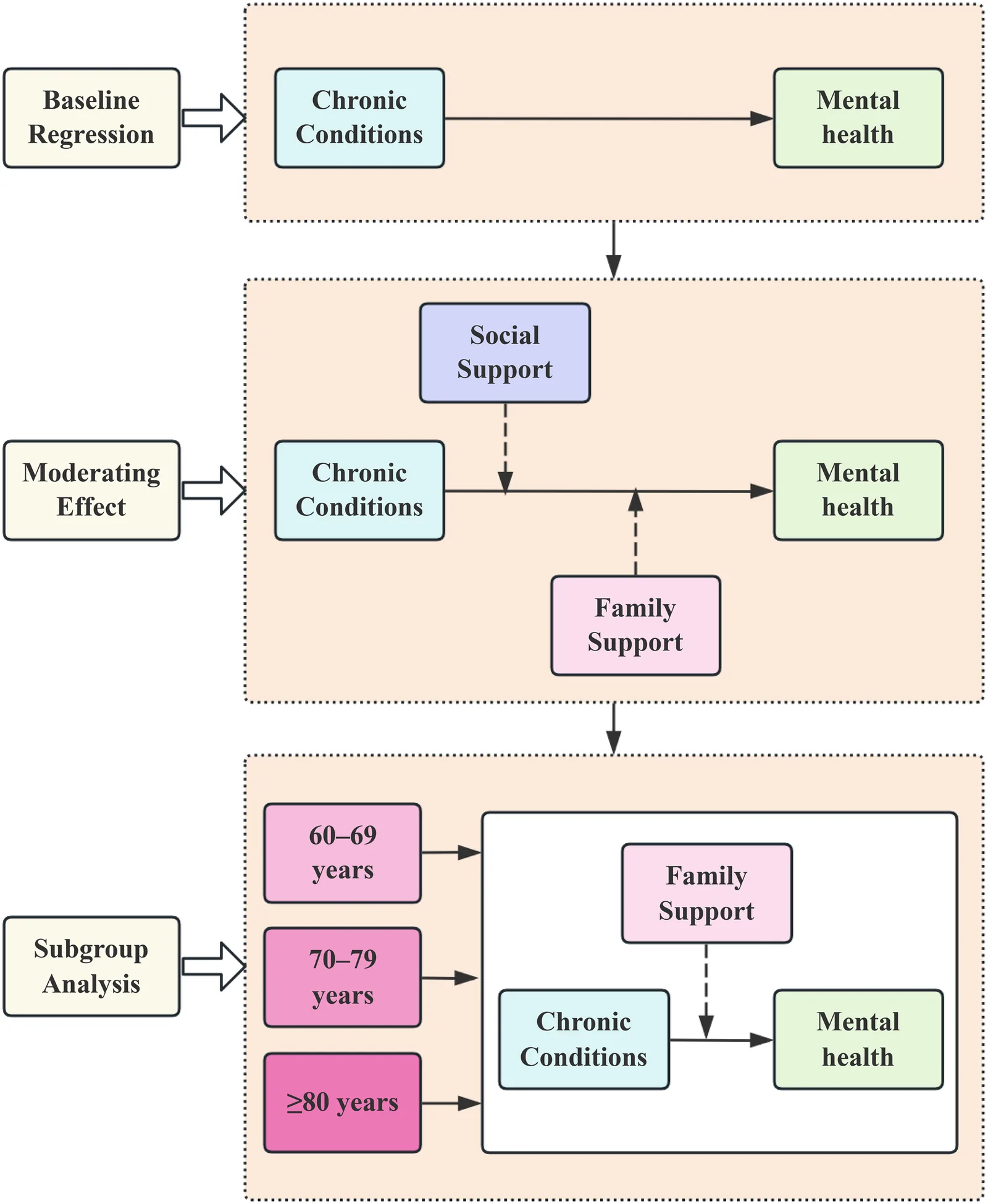
Analytical framework.

This study developed a sequential empirical framework (Fig 1) to systematically examine the association between multimorbidity and mental health among older adults and the underlying mechanisms. First, a baseline regression model was estimated to assess the direct association between multimorbidity and mental health. Building upon this analysis, social support and family support were introduced as moderating variables, and interaction models were estimated to examine whether these two forms of support differentially modify the association between multimorbidity and mental health. To further clarify the nature of the interaction effects, marginal effects analyses were conducted to estimate the association between multimorbidity and mental health at different levels of support, thereby illustrating how the relationship varies across support levels. Finally, to further examine whether the moderating role of family support differs across age groups, age-stratified regression analyses were conducted for three age groups (60–69 years, 70–79 years, and ≥80 years). This approach enabled us to assess variations in the moderating association of family support across different age groups in later life. Overall, the analytical framework follows a logical progression from main-effect estimation to moderation analysis, marginal-effects interpretation, and age-stratified regression analysis, providing a comprehensive assessment of the relationships among multimorbidity, support systems, and mental health in later life.

## Materials and methods

### Data sources

This study utilizes data from the fourth wave of the China Longitudinal Aging Social Survey (CLASS), conducted in 2020 by the Institute of Gerontology at Renmin University of China. CLASS is a nationally representative, longitudinal social survey that began with a baseline survey in 2014, followed by three follow-up waves in 2016, 2018, and 2020. The survey primarily targets older adults aged 60 and above within households, covering 28 provinces across China. The 2020 wave, the most recent follow-up, included 11,398 respondents. After excluding cases with missing values on key variables, the final analytical sample comprised 9,665 participants.

### Methods

The study involving human participants was approved by the Ethics Committee of the National Survey Research Center, Renmin University of China. The study was conducted in accordance with local legislation and institutional requirements. Written informed consent for participation was not required from the participants or their legal guardians/next of kin in accordance with national legislation and institutional requirements.

This study focuses on assessing the impact of chronic diseases on the mental health of older adults. By constructing a multidimensional indicator system and utilizing Stata 18 software, univariate descriptive statistical analysis was conducted, and regression models were developed to examine the relationships between the explanatory and dependent variables. In the empirical analysis, the Ordinary Least Squares (OLS) model was employed to evaluate the relationship between the comorbidity burden of chronic diseases and the mental health of older adults. Further analyses were conducted to enhance the robustness and credibility of the empirical findings.

This paper establishes the following baseline regression model:


$$\begin{array}{c} Y=\beta_{\text{0}}+\beta_{\text{1}}Dis+\beta_{i}X_{i}+\varepsilon_{\text{1}} \end{array}$$ (1)

where Y is the dependent variable, Dis is the key explanatory variable, $X_{i}$ is the vector of control variables, $\beta_{\text{0}}$ is the constant term, $\beta_{\text{1}}$ is the coefficient of key explanatory variable, $\beta_{i}$ is the coefficient of control variables, and $\varepsilon_{\text{1}}$ represents the error term.

To further examine the underlying mechanism, a moderation model is introduced. The specification is as follows:


$$\begin{array}{c} Y=\beta_{\text{0}}^{\prime }+\beta_{\text{1}}^{\prime }Dis+\alpha M+\delta Dis\times M+\beta_{i}^{\prime }X_{i}+\varepsilon_{\text{2}} \end{array}$$ (2)

where M is the mediating variable, Dis is the key explanatory variable, Dis × M is interaction term between the core explanatory variable and the moderating variable, $X_{i}$ is the vector of control variables, $\beta_{\text{0}}^{\prime }$ are constants, $\beta_{\text{1}}^{\prime }$ are the coefficients of key explanatory variable, δ is the coefficient of the interaction term. $\beta_{i}^{\prime }$ are the coefficients of control variables, and $\varepsilon_{\text{2}}$ represent error terms.

### Variable construction

#### Dependent variable.

The dependent variable in this study is mental health, which refers to the behavioral and cognitive manifestations of older adults’ psychological well-being, encompassing both positive and negative mental states. The Center for Epidemiologic Studies Depression Scale (CES-D), as revised by Silverstein et al., is widely recognized as a reliable instrument for assessing mental health among older adults. The scale comprises nine items assessing mood, life attitudes, appetite, sleep, and related aspects. Responses are coded on a three-point scale (“never,” “sometimes,” “often”) with scores ranging from 1 to 3. Negative items were reverse-coded, and the total score ranges from 9 to 27, with higher scores indicating lower levels of depressive symptoms and better mental health. In this study, the CES-D achieved a Cronbach’s alpha of 0.695, indicating acceptable internal consistency [21,22]. The details of the indicators are presented in Table 1.

**Table 1:** Dependent variable definitions and measurement.

Variable	Dimension	Item Code	Survey Question	Coding Scheme
Mental Health (MH)	Emotional Status	MH1	During the past week, did you feel in a good mood?	No = 1; Sometimes = 2; Often = 3
		MH2	During the past week, did you feel lonely?	Often = 1; Sometimes = 2; Never = 3
		MH3	During the past week, did you feel sad or depressed?	Often = 1; Sometimes = 2; Never = 3
	Life Satisfaction	MH4	During the past week, did you feel that your life was going well?	Often = 1; Sometimes = 2; Never = 3
		MH5	During the past week, did you feel that life was enjoyable or interesting?	No = 1; Sometimes = 2; Often = 3
	Psychosomatic Symptoms	MH6	During the past week, did you experience a loss of appetite?	Often = 1; Sometimes = 2; Never = 3
		MH7	During the past week, did you have difficulty sleeping?	Often = 1; Sometimes = 2; Never = 3
	Self-perception	MH8	During the past week, did you feel that you were useless?	Often = 1; Sometimes = 2; Never = 3
		MH9	During the past week, did you feel that you had nothing to do?	Often = 1; Sometimes = 2; Never = 3

Higher scores indicate better mental health outcomes after reverse coding of negatively worded items.

#### Key independent variable.

Multimorbidity burden among older adults. Multimorbidity was measured using a self-reported checklist of chronic conditions, including: hypertension; heart disease (e.g., coronary heart disease, congestive heart failure); diabetes or elevated blood glucose (including impaired glucose tolerance and fasting hyperglycemia); cerebrovascular disease (including stroke); kidney disease (excluding tumors or cancer); liver disease (excluding fatty liver, tumors, or cancer); tuberculosis; cervical or lumbar spine disorders; arthritis (including rheumatoid arthritis) or rheumatic diseases; breast disease; reproductive system disease; prostate disease; urinary system disease; glaucoma or cataract; cancer/malignant tumors (excluding minor skin cancer); dementia; osteoporosis; chronic pulmonary diseases such as chronic bronchitis, emphysema, or cor pulmonale (excluding tumors or cancer); neurological disorders; gastroenteritis or other digestive system diseases; Parkinson’s disease; and hearing loss. This disease list, based on prior epidemiological research and national disease surveillance priorities, reflects the main chronic conditions of public health concern in China. Respondents reporting one or more of these conditions were coded as having multimorbidity, and the total number of chronic conditions was used to represent the multimorbidity burden, with higher values indicating greater disease burden. Respondents reporting none of these conditions were coded as having no chronic disease [23,24].

#### Moderating variable.

Family support and social support were included as two separate moderating variables to capture support resources at different levels and their potential buffering effects. Family support was measured across two dimensions: family structure and family functional support, comprising four indicators. First, family structure was represented by the number of children, which serves as a foundational indicator of the potential resource pool and structural scale of the family network. Second, functional family support included three measures: frequency of social interactions, size of the emotional support network, and size of the instrumental support network. These indicators captured respondents’ contact with family or relatives, the number of family members available for emotional exchange, and the number of family members able to provide practical assistance when needed. An entropy-based method was applied to aggregate these indicators into a composite family support index, with higher values indicating greater family support [25,26].

Social support was measured across three dimensions—friend support, community support, and social participation—using a total of nine indicators. Friend support included frequency of social interactions, size of emotional support network, and size of instrumental support network, capturing the emotional and practical support obtained from friends. Community support focused on the availability of formal support resources, including whether the community provides home visit services, elderly hotlines, and psychological counseling, measured as binary variables. Social participation was measured by the frequency of participation in senior universities, leisure activities (e.g., Mahjong or chess), and group exercise activities (e.g., square dancing) over the past year, reflecting the level of social integration and participatory resources available to the individual [27,28]. The details of the indicators are presented in Table 2.

**Table 2:** Moderating variable definitions and measurement.

Variable	Dimension	Item Code	Survey Question	Coding Scheme
Family Support (FS)	Number of Children	FS1	How many children do you have (including biological, adopted, foster children, and those who have passed away)?	Count (number)
	Frequency of Family Interaction	FS2	How many family members or relatives do you meet or contact at least once a month?	None (0 persons) = 0; 1 person = 1; 2 persons = 2; 3–4 persons = 3; 5–8 persons = 4; ≥ 9 persons = 5
	Emotional Support Network Size	FS3	With how many family members or relatives can you comfortably discuss your private matters?	None (0 persons) = 0; 1 person = 1; 2 persons = 2; 3–4 persons = 3; 5–8 persons = 4; ≥ 9 persons = 5
	Instrumental Support Network Size	FS4	When you are in need, how many family members can provide you with help?	None (0 persons) = 0; 1 person = 1; 2 persons = 2; 3–4 persons = 3; 5–8 persons = 4; ≥ 9 persons = 5
Social Support (SS)	Friend Support	SS1	How many friends do you meet or contact at least once a month?	None (0 persons) = 0; 1 person = 1; 2 persons = 2; 3–4 persons = 3; 5–8 persons = 4; ≥ 9 persons = 5
		SS2	With how many friends can you comfortably discuss your private matters?	None (0 persons) = 0; 1 person = 1; 2 persons = 2; 3–4 persons = 3; 5–8 persons = 4; ≥ 9 persons = 5
		SS3	When you are in need, how many friends can provide you with help?	None (0 persons) = 0; 1 person = 1; 2 persons = 2; 3–4 persons = 3; 5–8 persons = 4; ≥ 9 persons = 5
	Community Support	SS4	Does your community provide home-based services?	No = 0; Yes = 1
		SS5	Does your community provide a hotline service for older adults?	No = 0; Yes = 1
		SS6	Does your community provide psychological counseling services?	No = 0; Yes = 1
	Social Participation	SS7	In the past year, how often did you participate in activities at universities for older adults?	Never = 0; A few times a year = 1; At least once a month = 2; At least once a week = 3; Almost daily = 4
		SS8	In the past year, how often did you participate in mahjong or chess activities?	Never = 0; A few times a year = 1; At least once a month = 2; At least once a week = 3; Almost daily = 4
		SS9	In the past year, how often did you participate in square dancing activities?	Never = 0; A few times a year = 1; At least once a month = 2; At least once a week = 3; Almost daily = 4

Higher values indicate stronger family or social support.

#### Control variable.

To minimize confounding due to individual differences, a set of control variables was included, covering demographic characteristics, socioeconomic status, and health and living conditions. Demographic characteristics included age (log-transformed, lnage), educational attainment, and marital status. Socioeconomic status was captured by annual personal income (log-transformed, lnincome) and number of properties owned. Health and living conditions included self-rated health, the need for assistance in daily life, and life satisfaction. These controls help account for differences in individual resource endowment, baseline health, and subjective life evaluation, thereby enhancing the robustness of the model estimates. Detailed descriptions of the variables are provided in Table 3.

**Table 3:** Control variable definitions and measurement.

Variable	Dimension	Symbol	Survey Question	Coding/ Measurement
Multimorbidity Burden	Number of Chronic Diseases	Dis	The number of chronic diseases reported by the respondent (the list of chronic conditions is provided in the main text).	Count (number)
Basic Characteristics	Age	Lnage	What is your age?	Years (log-transformed)
	Annual Personal Income	Lnincome	What was your total personal income in the past 12 months?	RMB (log-transformed)
	Education Level	Edu	What is your highest level of education attained?	0 = Illiterate; 6 = Primary school/ literacy class; 12 = Junior high school; 15 = Senior high school or vocational school; 18 = College and above
	Marital Status	Marital	What is your current marital status?	0 = Never married; 1 = Divorced; 2 = Widowed; 3 = Married (with spouse)
	Self-rated Health	Health	How would you rate your current health status?	Very unhealthy = 1; Unhealthy = 2; Fair = 3; Healthy = 4; Very healthy = 5
	Need for Care	Care	Do you currently need assistance with daily activities (e.g., eating, bathing, dressing, or using the toilet)?	No = 0; Yes = 1
	Life Satisfaction	Satis	Overall, how satisfied are you with your current life?	Very dissatisfied = 1; Dissatisfied = 2; Fair = 3; Satisfied = 4; Very satisfied = 5
	Number of Housing Units	House	How many housing units do you and your spouse own in total?	Count (number)

All income values are measured in Chinese yuan (RMB).

## Results

### Descriptive statistics

The mean number of chronic conditions among older adults was 1.686, indicating that most individuals had relatively few diseases, although a small proportion experienced multiple coexisting conditions, reflecting substantial individual variation. The mean level of family support was 0.405 with a standard deviation of 0.129, suggesting that, on average, older adults received a moderate-to-low level of family support, with a relatively concentrated distribution. Social support had a mean of 0.179 and a standard deviation of 0.147, indicating generally low levels of social support among most older adults, with a few individuals receiving higher support. Mental health scores among the elderly averaged 11.217 (SD = 3.265), reflecting a moderately high overall level of mental health in the sample, albeit with considerable individual differences. Descriptive statistics are presented in Table 4.

**Table 4:** Descriptive statistics.

Variable	N (Sample Size)	Maximum	Minimum	Mean	Standard Deviation (SD)	Median	Variance
Dis	9665	13	0	1.686	1.425	1	2.031
FS	9665	0.958	0	0.405	0.129	0.41	0.017
SS	9665	0.96	0	0.179	0.147	0.138	0.022
Y	9665	18	0	11.217	3.265	11	10.663
Lnage	9665	4.635	4.174	4.332	0.083	4.317	0.007
Lnincome	9665	12.919	0	3.648	4.411	0	19.454
Edu	9665	16	0	6.169	4.022	6	16.175
Marital	9665	3	0	2.735	0.501	3	0.251
Health	9665	5	1	3.405	0.897	3	0.804
Care	9665	1	0	0.063	0.242	0	0.059
Satis	9665	5	1	3.772	0.859	4	0.737
House	9665	5	0	1.063	0.388	1	0.151

### Baseline regression results

Table 5 presents the OLS regression results for the factors influencing mental health among older adults. To test the study hypotheses, nested models were estimated. Model 1 includes only control variables, such as demographic characteristics, socioeconomic status, health conditions, and family factors. Model 2 further incorporates the key independent variable, namely the number of chronic conditions. To examine potential multicollinearity issues, the Variance Inflation Factor (VIF) for all independent variables was calculated. The results show that all VIF values are well below 10, with a mean VIF of 1.19, indicating that multicollinearity is not a significant concern in this model.

**Table 5:** Baseline regression results.

	Model 1		Model 2	
Variable	Coefficient	Standard Error	Coefficient	Standard Error
Dis			−0.067***	0.023
Lnage	−3.370***	0.396	−3.212***	0.399
Edu	0.036***	0.009	0.034***	0.009
Marital	0.235***	0.063	0.233***	0.063
Health	0.547***	0.038	0.522***	0.039
Care	−0.548***	0.130	−0.535***	0.130
Lnincome	0.080***	0.008	0.083***	0.008
Satis	0.881***	0.039	0.877***	0.039
House	0.287***	0.081	0.311***	0.082
Constant	19.208***	1.783	18.713***	1.790
Adj. *R²*	0.164		0.165	
*F*	*F* = 231.91 *P* = 0.000***		F(9, 9655)=212.35 *P* = 0.000***	
Observations	9665		9665	

*** *p* < 0.01, ** *p* < 0.05, * *p* < 0.1. Adj. *R²* represents the adjusted coefficient of determination.

The *R*² of Model 1 is 0.164, which increases slightly to 0.165 in Model 2 after adding the key independent variable, indicating that the inclusion of multimorbidity modestly enhances the explanatory power of the model. Coefficients of the control variables remain largely stable across the two models, suggesting that the model specification is robust. Although the overall R² is relatively modest, this is common in studies of mental health outcomes, which are influenced by a wide range of individual, social, and environmental factors that are difficult to fully capture in survey-based analyses. Accordingly, Hypothesis 1 is supported.

After controlling for a range of socioeconomic, health, and family factors, Model 2 shows a significant negative association between the number of chronic conditions and mental health. This finding indicates that multimorbidity, as a major disease burden, exerts an independent and significant adverse effect on older adults’ mental health.

Regarding control variables, age is significantly negatively associated with mental health, whereas longer educational attainment, being married, better self-rated health, higher income, greater life satisfaction, and ownership of property are all positively associated with mental health. Conversely, requiring assistance with daily activities significantly reduces mental health, highlighting dependency on care as an important risk factor for psychological well-being.

### Moderating effect of social support

Table 6 presents the results of the moderation analysis examining the role of social support in the relationship between multimorbidity and mental health among older adults. Model 3 estimates the direct effects of multimorbidity, social support, and the control variables on mental health. Model 4 further includes the interaction term between multimorbidity and social support to assess the potential moderating effect of social support. To evaluate multicollinearity, a Variance Inflation Factor (VIF) test was conducted. All VIF values were well below the commonly accepted threshold of 10 (mean VIF = 1.63), indicating that multicollinearity is not a concern.

**Table 6:** Moderating effect of social support.

	Model 3		Model 4	
Variable	Coefficient	Standard Error	Coefficient	Standard Error
Dis	−0.043*	0.023	−0.063*	0.033
SS	2.310***	0.213	2.119***	0.317
Dis*SS			0.123	0.151
Lnage	−2.710***	0.400	−2.707***	0.400
Edu	0.026***	0.009	0.026***	0.009
Marital	0.233***	0.062	0.232***	0.062
Health	0.521***	0.039	0.522***	0.039
Care	−0.552***	0.129	−0.551***	0.129
Lnincome	0.073***	0.008	0.072***	0.008
Satis	0.860***	0.039	0.860***	0.039
House	0.285***	0.081	0.281***	0.081
Constant	16.267***	1.794	16.287***	1.794
Adj. *R²*	0.174		0.174	
*F*	*F*(10, 9654)=205.12, *P* = 0.000***		*F(*11, 9653) = 186.53, *P* = 0.000***	
Observations	9665		9665	

*** *p* < 0.01, ** *p* < 0.05, * *p* < 0.1. Adj. *R²* represents the adjusted coefficient of determination.

In Model 3, multimorbidity is negatively associated with mental health, whereas social support is positively associated with mental health. After the interaction term is introduced in Model 4, the negative association between multimorbidity and mental health remains statistically significant, and the positive effect of social support also remains robust. However, although the interaction coefficient between multimorbidity and social support is positive, it does not reach statistical significance. Therefore, there is no empirical evidence that social support significantly moderates the relationship between multimorbidity and mental health. Accordingly, Hypothesis 2 is not supported.

The coefficients and significance levels of the control variables remain largely consistent with those reported in the baseline model, indicating that the estimated model is robust. Overall, the findings suggest that social support contributes directly to better mental health among older adults but does not significantly alter the association between multimorbidity and mental health. These results imply that the beneficial role of social support is reflected primarily through its direct positive association with mental health rather than through the hypothesized stress-buffering mechanism.

**Fig 2:**
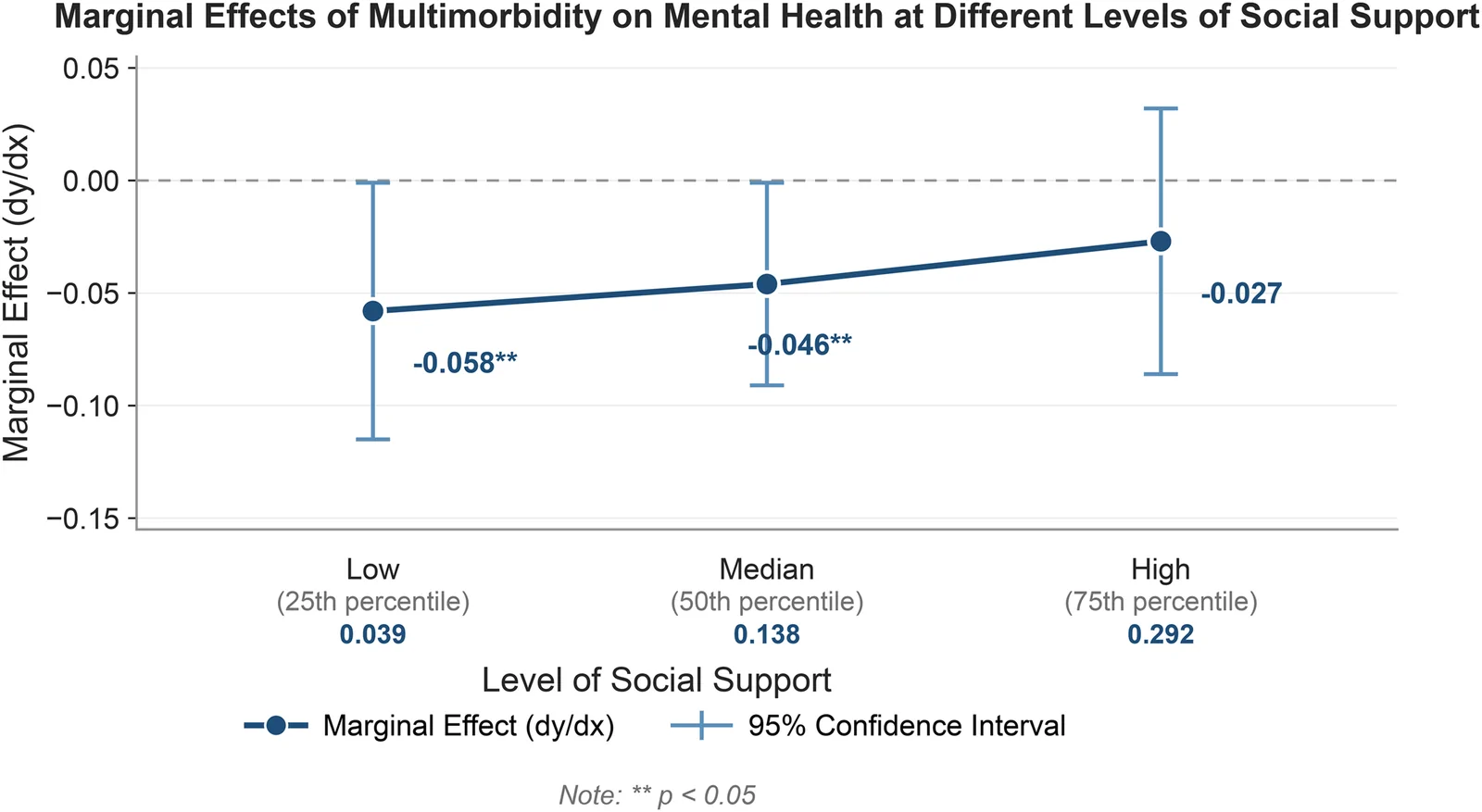
Marginal effects of chronic illness on mental health across representative levels of social support. The x-axis represents the level of social support, measured using three representative values corresponding to the 25th percentile (low level: 0.039), median (medium level: 0.138), and 75th percentile (high level: 0.292) of the social support distribution. The y-axis represents the estimated marginal effect of multimorbidity on mental health (dy/dx) based on the interaction model. The dots indicate the estimated marginal effects, and the vertical bars represent the corresponding 95% confidence intervals calculated using the delta method. Asterisks indicate statistical significance (*p < 0.05; **p < 0.01).

Table 7 and Fig 2 present the marginal effects of multimorbidity on mental health across different levels of social support. The results reveal a clear buffering pattern. Specifically, at low (25th percentile) and median (50th percentile) levels of social support, multimorbidity exerts a significant negative impact on mental health (dy/dx = −0.058, *p* < 0.05 and dy/dx = −0.046, *p* < 0.05, respectively). However, as social support reaches a high level (75th percentile), this negative marginal effect diminishes to −0.027 and loses statistical significance. Overall, these findings suggest a pattern of attenuation in the association between multimorbidity and mental health at higher levels of social support, although the moderating effect is not consistently statistically robust across the distribution.

**Table 7:** Marginal effects of multimorbidity on mental health at different levels of social support.

Level of Social Support	Representative Value	Marginal Effect (dy/dx)	Std. Err.	*t*	95% CI
Low (25th percentile)	0.039	−0.058**	0.029	−1.97	[-0.115, -0.000]
Median (50th percentile)	0.138	−0.046**	0.023	−1.98	[-0.091, -0.001]
High (75th percentile)	0.292	−0.027	0.030	−0.89	[-0.086, 0.032]

### Moderating effect of family support

Table 8 presents the results of the moderation analysis examining the role of family support in the relationship between multimorbidity and mental health among older adults. Model 5 evaluates the direct effects of chronic conditions, family support, and relevant control variables on mental health. Model 6 further incorporates the interaction term between chronic conditions and family support to test whether family support moderates the impact of multimorbidity on mental health.Subsequently, a Variance Inflation Factor (VIF) test was conducted. All VIF values were well below the threshold of 10 (mean VIF = 3.32), confirming that multicollinearity is not a significant concern in the model.

**Table 8:** Moderating effect of family support.

	Model 5		Meodel 6	
Variable	Coefficient	Standard Error	Coefficient	Standard Error
Dis	−0.070***	0.023	0.088	0.072
FS	1.230***	0.241	1.818***	0.350
Dis*FS			−0.381**	0.165
Lnage	−3.502***	0.403	−3.458***	0.403
Edu	0.034***	0.009	0.034***	0.009
Marital	0.200***	0.063	0.202***	0.063
Health	0.532***	0.039	0.532***	0.039
Care	−0.545***	0.130	−0.537***	0.130
Lnincome	0.085***	0.008	0.086***	0.008
Satis	0.868***	0.039	0.865***	0.039
House	0.296***	0.082	0.296***	0.082
Constant	19.574***	1.796	19.145***	1.805
Adj. *R²*	0.167		0.167	
*F*	*F(*10, 9654)= 194.22, *P =* 0.000***		*F*(10, 9654)= 177.13, *P* = 0.000***	
Observations	9665		9665	

*** *p* < 0.01, ** *p* < 0.05, * *p* < 0.1.Adj. *R²* represents the adjusted coefficient of determination.

In Model 5, multimorbidity is significantly and negatively associated with older adults’ mental health, whereas family support is significantly and positively associated with mental health. After including the interaction term in Model 6, the main effect of multimorbidity becomes statistically insignificant, while the positive association between family support and mental health remains robust. More importantly, the interaction term between multimorbidity and family support is negative and statistically significant at the 5% level, indicating that family support significantly moderates the association between multimorbidity and mental health. To further interpret the nature of this moderating effect, marginal effect analyses were subsequently conducted.

**Fig 3:**
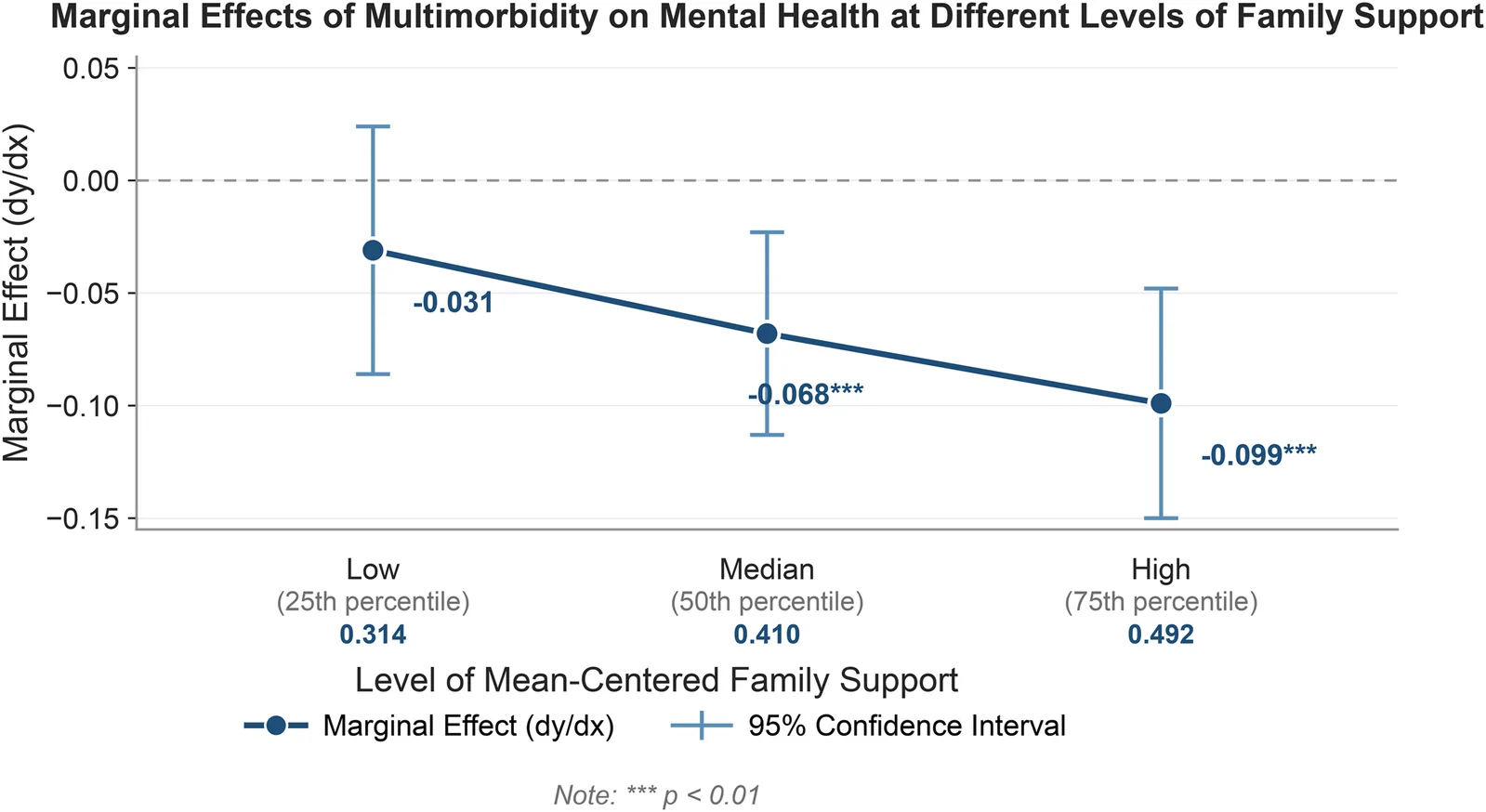
Marginal effects of chronic illness on mental health across representative levels of family support. The x-axis represents the level of family support, measured using three representative values corresponding to the 25th percentile (low level: 0.314), median (medium level: 0.410), and 75th percentile (high level: 0.492) of the family support distribution. The y-axis represents the estimated marginal effect of multimorbidity on mental health (dy/dx) at different levels of family support based on the interaction model. The dots indicate the estimated marginal effects, and the error bars represent the corresponding 95% confidence intervals calculated using the delta method. Asterisks indicate statistical significance (*p < 0.05; **p < 0.01).

To further interpret the moderating effect of family support, we estimated the marginal effect of chronic illness on mental health at three representative values of the family support variable corresponding to the 25th percentile (low family support, 0.314), the 50th percentile (median family support, 0.410), and the 75th percentile (high family support, 0.492). As presented in Table 9 and Fig 3, the estimated marginal effect of chronic illness became increasingly negative as the level of family support increased. Specifically, the marginal effect was −0.031 at the low level of family support and was not statistically significant. At the median level of family support, the marginal effect increased to −0.068, while at the high level of family support it further increased to −0.099. These findings indicate that the negative association between chronic illness and mental health becomes progressively stronger as family support increases, which is consistent with the significant interaction effect reported in Table 7.

**Table 9:** Marginal effects of chronic illness on mental health at representative levels of family support.

Level of Mean-Centered Family Support	Representative Value	Marginal Effect (dy/dx)	Std. Err.	*t*	95% CI
Low (25th percentile)	0.314	−0.031	0.028	−1.11	[−0.087, 0.024]
Median (50th percentile)	0.410	−0.068***	0.023	−2.98	[−0.113, -0.023]
High (75th percentile)	0.492	−0.099***	0.026	−3.82	[−0.150, -0.048]

### Subgroup analysis

The age-stratified regression analysis shows that the association between family support, mental health, and the moderating role of family support differs across age groups (Table 10). First, family support is positively associated with mental health across all age groups, with statistically significant coefficients for participants aged 60–69 years (*β* = 1.830, *p* < 0.05), 70–79 years (*β* = 1.583, *p* < 0.01), and 80 years and above (*β* = 1.834, *p* < 0.05). The magnitude of the association declines from the 60–69-year group to the 70–79-year group and then increases among participants aged 80 years and above.Second, the moderating effect of family support differs across age groups. The interaction term between multimorbidity and family support is significantly negative among participants aged 60–69 years (*β* = −1.215, *p* < 0.05) and 70–79 years (*β* = −0.476, *p* < 0.05), but is not statistically significant among those aged 80 years and above (*β* = −0.202, *p* > 0.05). Moreover, the magnitude of the interaction coefficient gradually decreases with age, indicating that the moderating association of family support becomes weaker among older age groups.

**Table 10:** Age-stratified analysis of the moderating effect of family support.

Variables	60–69 years	70–79 years	≥80 years
Dis	0.336 (0.219)	0.053 (0.097)	−0.062 (0.138)
FS	1.830** (0.916)	1.583*** (0.458)	1.834** (0.722)
Dis × Fs	−1.215 **(0.541)	−0.476** (0.226)	−0.202 (0.298)
Control variables	Yes	Yes	Yes
Constant	5.788*** (0.690)	5.298*** (0.359)	4.331*** (0.486)
Observations	1,259	5,704	2,702
Adj. *R²*	0.153	0.120	0.154

*** *p* < 0.01, ** *p* < 0.05, * *p* < 0.1. Adj. *R²* represents the adjusted coefficient of determination.

## Discussion

The baseline regression results indicate that a greater multimorbidity burden is associated with poorer mental health among older adults. This finding is consistent with previous studies showing that living with multiple chronic conditions is associated with a higher risk of psychological distress and poorer psychological well-being [29]. Multimorbidity often requires continuous disease management and long-term adaptation to physical limitations. These challenges may increase emotional stress and reduce psychological resilience over time. In China, rapid population aging and the growing prevalence of chronic diseases have further increased the complexity of disease management for older adults. Limited healthcare resources and gaps in long-term care services may also contribute to poorer mental health among individuals with multimorbidity [30,31].

The moderation and marginal effects analyses indicate that social support and family support exhibit distinct patterns in the relationship between multimorbidity and mental health. First, social support is positively associated with mental health, suggesting that older adults with greater social support generally report better psychological well-being [32]. However, the interaction between multimorbidity and social support is not statistically significant, indicating that the association between multimorbidity and mental health does not differ significantly across levels of social support. This finding may reflect the characteristics of the Chinese social support system. In the Chinese context, formal social support, such as community-based services and social activities, tends to provide broad and general forms of assistance, which may be less directly related to the long-term management of multimorbidity. Moreover, as noted by Fei Xiaotong in *From the Soil*, family relationships occupy the core of Chinese social networks, and formal social support often overlaps with family-based support, potentially reducing its independent role in modifying the association between multimorbidity and mental health [33].

Although the interaction term is not statistically significant, the marginal effects analysis reveals a descriptive pattern in which the estimated negative association between multimorbidity and mental health becomes numerically weaker as social support increases. From a descriptive perspective, the numerical weakening of the negative association at higher levels of social support aligns with the potential buffering role of external resources. For older adults with multimorbidity, high social support—such as community care, voluntary services, and emotional solidarity from peers—may provide a supportive environment. This environment may help alleviate the daily stress of managing chronic illnesses, thereby breaking the strong psychological link between physical frailty and mental distress [34].

Family support was also positively associated with mental health, suggesting that older adults receiving greater family support generally reported better psychological well-being. This finding is consistent with previous studies highlighting the important role of family members in providing emotional comfort, daily care, and a sense of security for older adults.The moderation analysis further showed that, after the interaction term was introduced, the coefficient for multimorbidity was no longer statistically significant, whereas family support remained positively associated with mental health and the interaction between multimorbidity and family support became statistically significant. These findings suggest that family support functions not only as an independent psychosocial resource associated with better mental health but also as a factor that shapes the association between multimorbidity and mental health [35,36].

To further clarify the nature of this interaction, marginal effects analyses were conducted. The results showed that the absolute magnitude of the marginal effect of multimorbidity on mental health increased as the level of family support increased, indicating that the negative association between multimorbidity and mental health became progressively stronger. Specifically, the association was not statistically significant among older adults reporting lower levels of family support but became statistically significant at moderate and high levels of family support.This finding is inconsistent with the protective effect predicted by the traditional stress-buffering hypothesis and therefore warrants cautious interpretation [37]. One possible explanation is that older adults with more severe health conditions and greater disease burden are more likely to receive intensive support and care from family members. Consequently, higher levels of family support may reflect a greater response to illness-related needs rather than a direct buffering effect against the adverse psychological consequences of multimorbidity. In other words, family support may be endogenous to disease severity, resulting in a closer correspondence between multimorbidity and family involvement.Another possible explanation can be drawn from psychological perspectives. Older adults living with multimorbidity may experience feelings of guilt, dependency, or loss of autonomy when they receive frequent assistance from family members. Although such support is well intentioned, intensive family involvement may inadvertently increase psychological burden by reinforcing individuals’ awareness of their declining health and their perceived caregiving burden on family members. As a result, greater family support may coincide with a stronger observed association between multimorbidity and poorer mental health [38,39].

The age-specific findings suggest that the role of family support differs across stages of later life. Although family support is positively associated with mental health in all age groups, its direct association follows a non-linear pattern. One possible explanation is that the relative importance of family support changes across the aging process. Younger-old adults generally maintain greater independence and broader social networks, allowing family support to complement other psychosocial resources. During the transition to ages 70–79 years, retirement adaptation, changing social roles, and gradual health decline may increase reliance on multiple support sources, thereby reducing the relative contribution of family support alone. Among the oldest-old, increasing physical frailty, functional limitations, and dependence on daily care may make family support a more central source of emotional and instrumental assistance, strengthening its positive association with mental health [40].

In contrast, the moderating role of family support gradually weakens with age and becomes statistically non-significant among adults aged 80 years and above. This finding suggests that, although family support remains an important psychosocial resource, its role in shaping the association between multimorbidity and mental health may diminish in advanced old age [41,42]. A possible explanation is that the psychological consequences of multimorbidity among the oldest-old are increasingly influenced by severe functional decline, frailty, and complex care needs, reducing the relative contribution of family support in modifying this association. These interpretations should be considered cautiously and warrant further investigation using longitudinal data.

## Conclusions

Based on the empirical findings of this study, several conclusions can be drawn. First, multimorbidity is significantly associated with poorer mental health among older adults, confirming the substantial psychological burden associated with chronic disease accumulation.Second, although both social support and family support are positively associated with mental health, they play different roles in the relationship between multimorbidity and mental health. Social support improves psychological well-being but does not significantly moderate the association between multimorbidity and mental health. In contrast, family support not only shows a positive direct association with mental health but also significantly moderates this relationship.Third, the marginal effects analysis indicates that the negative association between multimorbidity and mental health becomes progressively stronger as family support increases, suggesting that the role of family support is more complex than predicted by the traditional stress-buffering hypothesis.Finally, the subgroup analysis reveals distinct age-related patterns. The direct association between family support and mental health follows a U-shaped pattern across age groups, whereas the moderating effect of family support gradually weakens with age, remaining significant among adults aged 60–79 years but disappearing among those aged 80 years and above. These findings suggest that the direct and interactive associations of family support differ across stages of later life, highlighting the need for age-specific family-based strategies to support mental health among older adults with multimorbidity.

From a practical and policy perspective, these conclusions yield four specific implications for healthy aging strategies:

First, greater attention should be paid to the mental health of older adults living with multimorbidity. Current chronic disease management programs primarily emphasize physical health outcomes, whereas psychological well-being is often overlooked. Integrating routine mental health screening, psychological counseling, and emotional support into primary healthcare services could help reduce the psychological burden associated with multimorbidity.

Second, community-based social support systems should continue to be strengthened. Although social support did not significantly moderate the relationship between multimorbidity and mental health, it remained positively associated with better psychological well-being. Expanding community services, increasing opportunities for social participation, and improving access to age-friendly social resources may contribute to maintaining mental health among older adults.

Third, greater emphasis should be placed on improving the quality rather than simply the quantity of family support. The findings suggest that family support is positively associated with mental health but exhibits a complex interaction with multimorbidity. Policies should therefore provide guidance and training for family caregivers, improve family caregiving skills, and establish integrated support services that reduce caregiving burden while enhancing the effectiveness of family-based care.

Finally, interventions should adopt an age-specific approach. The subgroup analysis indicates that the direct association of family support with mental health follows a U-shaped pattern across age groups, whereas its moderating role gradually weakens with age. Accordingly, family-centered interventions may be particularly beneficial for younger-old adults, while the oldest-old are likely to require more comprehensive support involving healthcare services, long-term care, and community-based assistance in addition to family support.

## Limitations and future work

Although this study elucidates the associations between chronic disease burden, informal support systems, and mental health among older adults, several limitations should be acknowledged. First, the cross-sectional nature of the data restricts the ability to establish causal relationships. Future research could address this by utilizing longitudinal designs to track the dynamic interplay between chronic disease progression and psychological well-being over time.

Second, multimorbidity was operationalized using a simple count of self-reported chronic conditions, which does not account for clinical nuances such as disease severity, duration, or specific condition combinations. Future studies are encouraged to employ more refined multimorbidity measures to better assess how distinct clinical profiles are associated with mental health.

Third, the potential conceptual overlap between life satisfaction and mental health warrants caution. While these constructs are theoretically distinct—representing cognitive evaluations and affective states, respectively—their inherent correlation remains a methodological challenge. Future research could employ more refined psychometric assessments or structural equation modeling to better disentangle the complex interplay between these dimensions.

Finally, while the national representation of the CLASS dataset is significant, regional and cultural variations in China may influence the identified associations. Future studies could expand the scope of analysis to incorporate broader socio-cultural contexts and more granular categorizations of support types. Such efforts would provide a more nuanced understanding of the mechanisms of psychosocial adaptation, offering more robust empirical evidence for the development of mental health management strategies and policy interventions in aging societies.
